# Expression of *degQ* gene and its effect on lipopeptide production as well as formation of secretory proteases in *Bacillus subtilis* strains

**DOI:** 10.1002/mbo3.1241

**Published:** 2021-10-14

**Authors:** Lars Lilge, Maliheh Vahidinasab, Isabel Adiek, Philipp Becker, Chanthiya Kuppusamy Nesamani, Chantal Treinen, Mareen Hoffmann, Kambiz Morabbi Heravi, Marius Henkel, Rudolf Hausmann

**Affiliations:** ^1^ Department of Bioprocess Engineering (150k) Institute of Food Science and Biotechnology (150) University of Hohenheim Stuttgart Germany

**Keywords:** *Bacillus subtilis*, *degQ*, lipopeptide, plipastatin, secretory proteases, surfactin

## Abstract

*Bacillus subtilis* is described as a promising production strain for lipopeptides. In the case of *B*. *subtilis* strains JABs24 and DSM10^T^, surfactin and plipastatin are produced. Lipopeptide formation is controlled, among others, by the DegU response regulator. The activating phospho‐transfer by the DegS sensor kinase is stimulated by the pleiotropic regulator DegQ, resulting in enhanced DegU activation. In *B*.* subtilis* 168, a point mutation in the *degQ* promoter region leads to a reduction in gene expression. Corresponding reporter strains showed a 14‐fold reduced expression. This effect on *degQ* expression and the associated impact on lipopeptide formation was examined for *B*. *subtilis* JABs24, a lipopeptide‐producing derivative of strain 168, and *B*. *subtilis* wild‐type strain DSM10^T^, which has a native *degQ* expression. Based on the stimulatory effects of the DegU regulator on secretory protease formation, the impact of *degQ* expression on extracellular protease activity was additionally investigated. To follow the impact of *degQ*, a deletion mutant was constructed for DSM10^T^, while a natively expressed *degQ* version was integrated into strain JABs24. This allowed strain‐specific quantification of the stimulatory effect of *degQ* expression on plipastatin and the negative effect on surfactin production in strains JABs24 and DSM10^T^. While an unaffected *degQ* expression reduced surfactin production in JABs24 by about 25%, a sixfold increase in plipastatin was observed. In contrast, *degQ* deletion in DSM10^T^ increased surfactin titer by threefold but decreased plipastatin production by fivefold. In addition, although significant differences in extracellular protease activity were detected, no decrease in plipastatin and surfactin produced during cultivation was observed.

## Graphical abstract

The lipopeptide‐producing *Bacillus subtilis* strains JABs24 and DSM10^T^ differ by one single base substitution in the *degQ* promoter region, resulting in drastically reduced *degQ* expression in JABs24. Subsequent deletion of the *degQ* gene in DSM10^T^ or integration of a natively expressed *degQ* version in JABs24 revealed, among others, the differential effects of DegQ on the formation of the lipopeptides surfactin and plipastatin. Additional effects on the formation of secretory proteases do not seem to have any influence on lipopeptide stability.

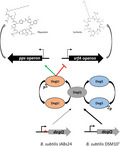

## 1 INTRODUCTION

*B*. *subtilis* is one of the best characterized gram‐positive bacteria and serves as a model organism for fundamental and applied research. The knowledge about the physiology of *B*. *subtilis* made this strain an important microbial host in biotechnology (Stein, 2005). In this context, *B*. *subtilis* is used as a super‐secreting cell factory due to benefits such as excellent fermentation properties, high product yields in gram per liter range, and the lack of toxic by‐products (van Dijl & Hecker, 2013). In addition to the production of industrially relevant enzymes and vitamins (Cui et al., 2018), *B*. *subtilis* natively forms a variety of secondary metabolites. Among these compounds, three lipopeptide families, in particular, namely surfactin, iturin, and fengycin, are reported to have broad bioactivity based on a common amphipathic structure comprising a fatty acid linked to a peptide moiety (Geissler et al., 2019; Marvasi et al., 2010). Different amino acid sequences in the circular peptide and variable fatty acid chain lengths give each lipopeptide unique properties (Zhao et al., 2017). In the genome of *Bacillus* spp., bacteria encoding for fengycin biosynthesis also show the ability to produce surfactin (Kim et al., 2010; Yaseen et al., 2018). In this context, regulatory crosstalk of non‐ribosomal peptide synthetases (NRPSs) is conceivable (Vahidinasab et al., 2020; Yaseen et al., 2018). Surfactin is described as one of the most powerful microbially produced biosurfactants and has great potential to be used in many industrial sectors such as cosmetics, pharmaceuticals, as well as food (Henkel et al., 2017; Hoffmann et al., 2021). The benefits of surfactin are not limited to emulsifying activity, as some studies reported antimicrobial and anticancer properties (Béven & Wroblewski, 1997; Kameda et al., 1974). Fengycins, including plipastatin as a member of this group, have been shown to have several antagonistic effects for soil‐borne fungal phytopathogens and may act as elicitors for systemic plant resistance (Cawoy et al., 2015). Moreover, fengycin has been described to have antiviral, antibacterial, and anticancer properties (Huang et al., 2006; Ongena et al., 2007; Raaijmakers et al., 2010; Yin et al., 2013). Due to these characteristics, fengycin has great potential for future agricultural applications.

Structurally, lipopeptides consist of a cyclic peptide and a fatty acid chain. In the case of surfactin and plipastatin, a hepta‐ or deca‐peptide moiety, respectively, is linked to a β‐hydroxy fatty acid chain of varying length (Cochrane & Vederas, 2016; Gao et al., 2018). The production of lipopeptides depends on NRPSs expressed by the *srfAA*‐*AD* operon for surfactin and by the *ppsA*‐*E* operon for plipastatin (Nakano et al., 1988; Tosato et al., 1997). Posttranslationally, NRPSs need to be activated by the 4‐phosphopantetheinyl transferase Sfp (Nakano et al., 1992; Quadri et al., 1998). In addition, superordinated stimuli such as quorum sensing and nutrient availability influence lipopeptide biosynthesis. Specifically, several global regulators including Spo0A, AbrB, CodY, and DegU are involved in the control of NRPS expression (Nakano et al., 1992; Serror & Sonenshein, 1996; Sun et al., 2021; Vahidinasab et al., 2020).

Different physiological adaptations are associated with the DegU regulon, including the formation of extracellular enzymes, genetic competence, and biofilm formation (Dahl et al., 1992; Kobayashi, 2007; Mäder et al., 2002; Msadek et al., 1990; Shimotsu & Henner, 1986). Moreover, also surfactin and plipastatin production are affected by DegU regulation (Miras & Dubnau, 2016; Tsuge et al., 1999). As a response regulator, DegU is part of the two‐component DegS‐DegU system. After activating phospho‐transfer from histidine kinase DegS to the response regulator DegU, the phosphorylated DegU version (DegU‐P) can regulate the expression of various genes (Murray et al., 2009). In addition to this process, DegQ, a small protein of 46 amino acids, stimulates the autophosphorylation of DegS and is important for the complete activation of DegU by phosphorylation (Do et al., 2011; Yang et al., 1986). In the case of the domesticated laboratory model strain *B*.* subtilis* 168, a single base mutation in the −10 box silences *degQ* gene expression (Stanley & Lazazzera, 2005). As a result, phospho‐transfer for DegU activation is reduced.

In this study, the lipopeptide‐producing *B*. *subtilis* strain JABs24, an *sfp* ^+^ derivative of *B*.* subtilis* 168, and the wild‐type strain DSM10^T^, which exhibits a native *degQ* expression, were used to analyze the effect of *degQ* expression on lipopeptide production and formation of secretory proteases.

## 2 MATERIALS AND METHODS

### 2.1 Chemicals, materials, and standard procedures

All chemicals were purchased from Carl Roth GmbH & Co. KG, if not otherwise mentioned. Standard molecular techniques were performed as described by Sambrook and Russell (2006). PCRs were carried out on a PCR thermal cycler (peqSTAR 96X VWR GmbH) using DNA polymerase (Phusion High‐Fidelity #M0530S, New England BioLabs). PCR reactions were purified after agarose‐based gel electrophoresis using QIAquick PCR & Gel Cleanup Kit (Qiagen). Plasmid DNA was extracted with innuPREP Plasmid Mini Kit (Analytik Jena AG), and chromosomal DNA was purified using the ready‐to‐use innuPREP Bacteria DNA Kit (Analytik Jena AG) according to the manufacture's instruction.

### 2.2 Strain construction, plasmids, and transformation method

All strains and plasmids used in this study are summarized in Table 1. The oligonucleotides used to construct the strains and plasmids are listed in Table 2. *Escherichia coli* JM109 was used for plasmid propagation and cloning. Transformation of naturally competent *B*. *subtilis* strains was performed according to the “Paris method” (Harwood & Cutting, 1990). Depending on the selection marker, transformants were selected on Lysogeny Broth agar supplemented with ampicillin (100 µg/ml), spectinomycin (100 µg/ml), or erythromycin (10 µg/ml for *E*. *coli* and 5 µg/ml for *B*. *subtilis*). All plates were incubated at 37℃.

**TABLE 1:** Bacterial strains and plasmids used in this study

Strain or plasmid	Origin or Genotype	References
Strains		
*Escherichia coli*		
JM109	*mcrA recA1 supE44 endA1 hsdR17* (*r*_K_^−^ *m*_K_^+^)	Yanisch‐Perron et al. (1985)
	*gyrA96 relA1 thi* Δ(*lac*‐*proAB*)	
	*F*´[*traD36 proAB* ^+^ *lacI* ^q^ *lacZ* Δ*M15*]	
*Bacillus subtilis*		
JABs24	*trp*+; Δ*manPA*; *sfp*+	Geissler et al. (2019)
DSM10^T^	wild‐type strain	German Collection of Microorganisms and Cell Cultures
		GmbH
BCKN1	*trp*+; Δ*manPA*; *sfp*+;	This study
	Δ*amyE*::+510 bp‐*degQ*	
BCKN2	DSM10^T^; Δ*degQ*::*erm*	This study
BKE31720	*trpC2*; Δ*degQ*::*erm*	Bacillus Genetic Stock Center
BMV15	DSM10^T^ wild‐type;	This study
	*amyE*::[P*_degQ_*‐*lacZ*, *spcR*]	
	(*degQ* promoter region for *lacZ* fusion	
	was derived from *B*. *subtilis* DSM10^T^)	
BMV16	*trp*+; Δ*manPA*; *sfp*+;	This study
	*amyE*::[P*_degQ_*‐*lacZ*, *spcR*]	
	(*degQ* promoter region for *lacZ* fusion	
	was derived from *B*. *subtilis* DSM10^T^)	
BMV17	DSM10^T^ wild‐type;	This study
	*amyE*::[P*_degQ_*‐*lacZ*, *spcR*]	
	(*degQ* promoter region for *lacZ* fusion	
	was derived from *B*. *subtilis* JABs24)	
BMV18	*trp*+; Δ*manPA*; *sfp*+;	This study
	*amyE*::[P*_degQ_*‐*lacZ*, *spcR*]	
	(*degQ* promoter region for *lacZ* fusion	
	was derived from *B*. *subtilis* JABs24)	
Plasmids		
pKAM446	*ori*_pUC18_, *bla*, *rop*, *ermC*,	Hoffmann et al. (2021)
	*amyE*′‐[*ter*‐P*_srfAA_*‐*lacZ*, *spcR*]‐ ′*amyE*	
pMAV5	*ori*_pBR322_, *rop*, *ermC*, *bla*,	Vahidinasab et al. (2020)
	*amyE*´‐[*ter*‐P*_glcR_* ‐ +510 bp‐*degQ*‐*spcR*]‐´*amyE*	
pMAV14	*ori*_pUC18_, *bla*, *rop*, *ermC*,	This study
	*amyE*′‐[*ter*‐P*_degQ_*‐*lacZ*, *spcR*]‐ ′*amyE*	
	(*degQ* promoter sequence	
	derived from *B*. *subtilis* JABs24)	
pMAV15	*ori*_pUC18_, *bla*, *rop*, *ermC*,	This study
	*amyE*′‐[*ter*‐P*_degQ_*‐*lacZ*, *spcR*]‐ ′*amyE*	
	(*degQ* promoter sequence	
	derived from *B*. *subtilis* DSM10^T^)	

**TABLE 2:** Oligonucleotides used in this study

Primer	Sequence (5´→ 3´)	Purpose
S1411	GATTAAAGACCGTATCCACTTC	Amplification of
S1412	GGCGCTTAAGATATAAGTAAATCAG	Δ*degQ*::*erm* locus from BKE31720 strain
S1079	TCGGTGAAAAATGAGCC	Verification of Δ*degQ*::*erm* integration into DSM10^T^ strain
S1080	GCTCAATAACGACTTCC	
S1009	CTGCCGTTATTCGCTGGATT	Verification of +510 bp‐*degQ* integration into *amyE* locus
S1010	AGAGAACCGCTTAAGCCCGA	
S1699	TGGATCCGGCGCCCACGTGGCTCG‐	Construction of P*_degQ_* reporter plasmids
	CAAAAAAGGATGTTTCTATATG	
S1700	AGTGAATCCGTAATCATGGTCATCG‐	
	TTTCCACACTCCTTT	

For the construction of BCKN1, the *degQ* gene of *B*. *subtilis* DSM10^T^ including native promoter region (+ 510 bp) and terminator structure was integrated into the *amyE* locus of *B*. *subtilis* JABs24 using plasmid pMAV5 (Vahidinasab et al., 2020). BCKN2 was created by integrating the deletion of *degQ* gene in *B*. *subtilis* DSM10^T^ using chromosomal DNA of *Bacillus* knockout erythromycin (BKE) strain BKE31720 carrying the deletion of the *degQ* gene (Δ*degQ*::*erm*) (Koo et al., 2017). The plasmids for the construction of the P*_degQ_* reporter strains were cloned using Gibson Assembly (New England BioLabs). Therefore, the pKAM446 plasmid was digested with *Nhe*I and *Nde*I before integrating amplified *degQ* promoter regions from JABs24 and DSM10^T^, respectively. The correctness of all mutant strains was ensured by sequencing (Eurofins Genomics Germany GmbH).

### 2.3 Cultivation and growth conditions

The composition of the mineral salt medium used in this study was based on the fermentation medium containing 8 g/L glucose of Willenbacher et al., (2014) with slight modifications: 4.0 × 10^−6^ M Na_2_EDTA ×2 H_2_O, 7.0 × 10^−6^ M CaCl_2_, 4.0 × 10^−6^ M FeSO_4_ × 7 H_2_O, 1.0 × 10^−6^ M MnSO_4_ × H_2_O, 50 mM (NH_4_)_2_SO_4_, 0.03 M KH_2_PO_4_, 0.04 M Na_2_HPO_4_ × 2 H_2_O and 8.0 × 10^−4^ M MgSO_4_ × 7 H_2_O. An overnight culture in Lysogeny Broth medium (10 g/L tryptone, 5 g/L NaCl, 5 g/L yeast extract) was used for the first preculture. The second preculture using a mineral salt medium was inoculated with exponentially growing cells from the first preculture. When the cell culture reached the exponential phase, the main culture was inoculated into 1 L Erlenmeyer baffled flasks with a final volume of 100 ml and an initial OD_600_ of 0.1. All cultivations had three biological replicates and were performed at 37℃ and 0.4 *g* in an incubation shaker (Innova 44^®^R, Eppendorf AG). Samples were taken regularly every four hours to measure optical density (OD_600_) using a spectrophotometer (Biochrom WPA CO8000, Biochrom Ltd.), glucose concentration using HPTLC measurement (Geissler et al., 2019), β‐galactosidase activity (Miller Assay) described by Hoffmann et al., (2020), and (endo)‐protease activity. Surfactin and plipastatin concentration were measured as previously described by Geissler et al., (2017). Specifically, 2 ml of cell‐free supernatant was extracted three times with chloroform/methanol (2:1). The pooled solvent layers were dried using a rotary evaporator at 10 mbar and 40℃. Dried samples were resolved in 2 ml methanol and applied in 6 mm bands on a silica HPTLC plate. As a mobile phase, chloroform/methanol/water (65:25:4) was used with a migration distance over 60 mm. Surfactin standard from Sigma Aldrich and plipastatin standard from Lipofabrik were used for quantification.

### 2.4 Data analysis

For the conversion of OD_600_ into cell dry weight (CDW), the correlation factor of 3.762 was determined in a preliminary experiment described by Willenbacher et al. (2014). The product yield of biomass *Y_P_*_/_ *_X_* [g g^−1^] was calculated using Equation 1. For the calculation, the mean values of the total mass of the product (m_Surfactin_, m_Plipastatin_) and CDW (m_CDW_) from the beginning of cultivation to the time point at the end of the exponential phase were used.

(1) $$Y_{P/X}=\frac{{\Delta m}_{\mathrm{surfactin}\;\mathrm{or}\;\mathrm{plipastatin}}}{m_{\mathrm{CDW}}}\;\left[g∙g^{‐1}\right]$$

### 2.5 Assay for extracellular protease activity

The total activity of the degrading proteins in the cultivation medium was analyzed by azocasein assay. The measurement method was adapted from Charney and Tomarell (1947) and applied by Baur et al., 2015. In detail, 100 µl of cell‐free supernatant was mixed with an equal volume of a pre‐warmed (40℃ for 5 min) azocasein solution (5 g/L, pH 7, dissolved in H_2_O_dd_) and subsequently incubated for 1 h at 37℃ and 1.07 *g*. The reaction was stopped by adding 20 µl trichloroacetic acid (2 M). Precipitated azocasein was removed by centrifugation at 1715 *g* for 10 min at 4℃. Subsequently, 150 µl of the supernatant was transferred to a microtiter plate and mixed with 50 µl NaOH (1 M). The absorbance was measured in a microtiter plate spectrophotometer (MULTISKAN GO, Thermo Scientific) at 450 nm. A blank for the measurement was performed with the cell‐free supernatant after the addition of trichloroacetic acid. A calculation of the protease activity is summarized in Equations (2) and (3).

(2) $$\Delta A=\frac{\sum A}{n}‐A_{\mathrm{Blank}}\left[‐\right]$$

(3) $$EA=\frac{\Delta A∙F∙V}{t∙v}∙10\;\left[\Delta A∙h^{‐1}∙{\mathrm{mL}}^{‐1}\right]$$

The calculation of the absorption difference (ΔA) is described in Equation (2). The following volumetric peptidase activity is defined in Equation (3) and is determined by the absorption difference (ΔA), a dilution factor (F), the total measurement volume (V) [µl], the incubation time (t) [h], and the volume of the cell‐free supernatant used for the assay (v) [µl].

## 3 RESULTS

### 3.1 Expression of *degQ* gene in *B. subtilis* strains JABs24 and DSM10^T^

**FIGURE 1:**
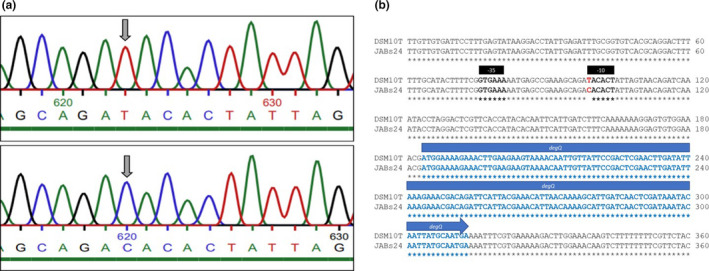
Comparison of *degQ* locus between JABs24 (168 *sfp*+) and DSM10^T^ strain. (a) The chromatograms obtained after the sequencing process show the base‐pair substitution (T::C) in the −10 promoter region of *degQ*. The extended *degQ* regions of *B*.* subtilis* strains DSM10^T^ (top) and JABs24 (bottom) were amplified and sequenced by Eurofins Genomics (Ebersberg, Germany). (b) Obtained sequences were compared and identical nucleotides were marked by stars (*). Information about the annotation of the *degQ* promoter region was used from Stanley and Lazazzera (2005)

As a derivative of *B*. *subtilis* 168, the surfactin‐forming strain JABs24 exhibits a point mutation within the −10 box of the *degQ* gene compared to *B*. *subtilis* wild‐type strains such as *B*. *subtilis* DSM10^T^ (Figure 1). This base substitution (T::C) in the promoter region of *degQ* was previously described by Stanley and Lazazzera (2005) and leads to significantly reduced gene expression of *degQ* in strain 168. This point mutation is also prominent when comparing the genome sequences of *B*. *subtilis* strains 168 and DSM10^T^ (Kunst et al., 1997; Lilge et al., 2021).

**FIGURE 2:**
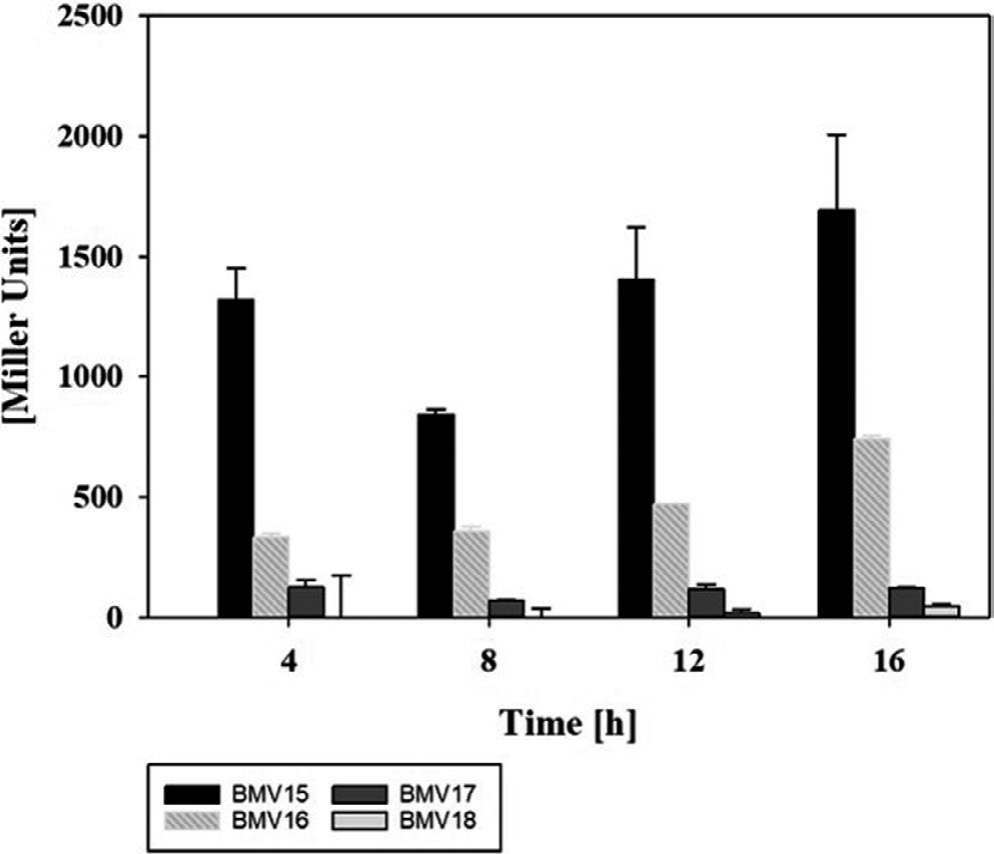
Comparison of *degQ* gene expression under the control of native and point‐mutated *degQ* promoter during 16‐hour shake flask cultivation with 8 g/L glucose. The *lacZ* fusion construct with native *degQ* promoter was chromosomally integrated into *B*. *subtilis* DSM10^T^ and JABs24, resulting in strains BMV15 and BMV16, respectively. Similarly, strains BMV17 and BMV18 are the reporter strains with point‐mutated *degQ* promoter for DSM10^T^ and JABs24. Data points represent a mean of three biological replicates. The error bars show the standard deviation of the calculated values

To analyze the effect of promoter point mutation on *degQ* gene expression, reporter strains with chromosomally integrated P*_degQ_*‐*lacZ* fusions were constructed. Accordingly, time‐resolved expression patterns were measured for both *degQ* promoter versions in JABs24 (BMV16 and BMV18) and DSM10^T^ (BMV15 and BMV17). The corresponding β‐galactosidase activity showed that the P*_degQ_* promoter region of DSM10^T^ exhibited a significantly higher expression level compared with that of JABs24 (Figure 2). In the transition between the exponential and stationary phase, approx. 14‐fold higher Miller Units were detected for the *lacZ* fusion with *degQ* promoter from DSM10^T^.

In addition to the confirmation that *degQ* expression is nearly silenced in strain JABs24, the results have shown that both *degQ* promoter versions were more active in the DSM10^T^ background. Thus, threefold increased promoter activity was detected for P*_degQ_* ^DSM10T^ and sixfold higher Miller Units were calculated for P*_degQ_* ^JABs24^ in the transition phase.

### 3.2 Effect of *degQ* expression on the formation of lipopeptides and secretory proteases

It is already known that DegQ acts as a stimulator for autophosphorylation of DegS signal kinase leading to enhanced activation of DegU response regulator (Do et al., 2011). In the active state, DegU‐P controls a variety of genes encoding secretory proteases, flagellin proteins, and non‐ribosomal peptide synthetases for the biosynthesis of plipastatin and surfactin (Hsueh et al., 2011; Mäder et al., 2002; Miras & Dubnau, 2016; Tsuge et al., 1999; Wang et al., 2015). To get an overview of the influence of different *degQ* gene expressions on biotechnologically relevant production of plipastatin and surfactin as well as secretory proteases, production strains with different *degQ* expression capabilities were analyzed. For this purpose, the wild‐type strains JABs24, DSM10^T^, and their *degQ* mutant strains BCKN1 and BCKN2 were examined. While BCKN1 represents strain JABs24 with native *degQ* gene expression, BCKN2 is the DSM10^T^ strain with *degQ* deletion.

**FIGURE 3:**
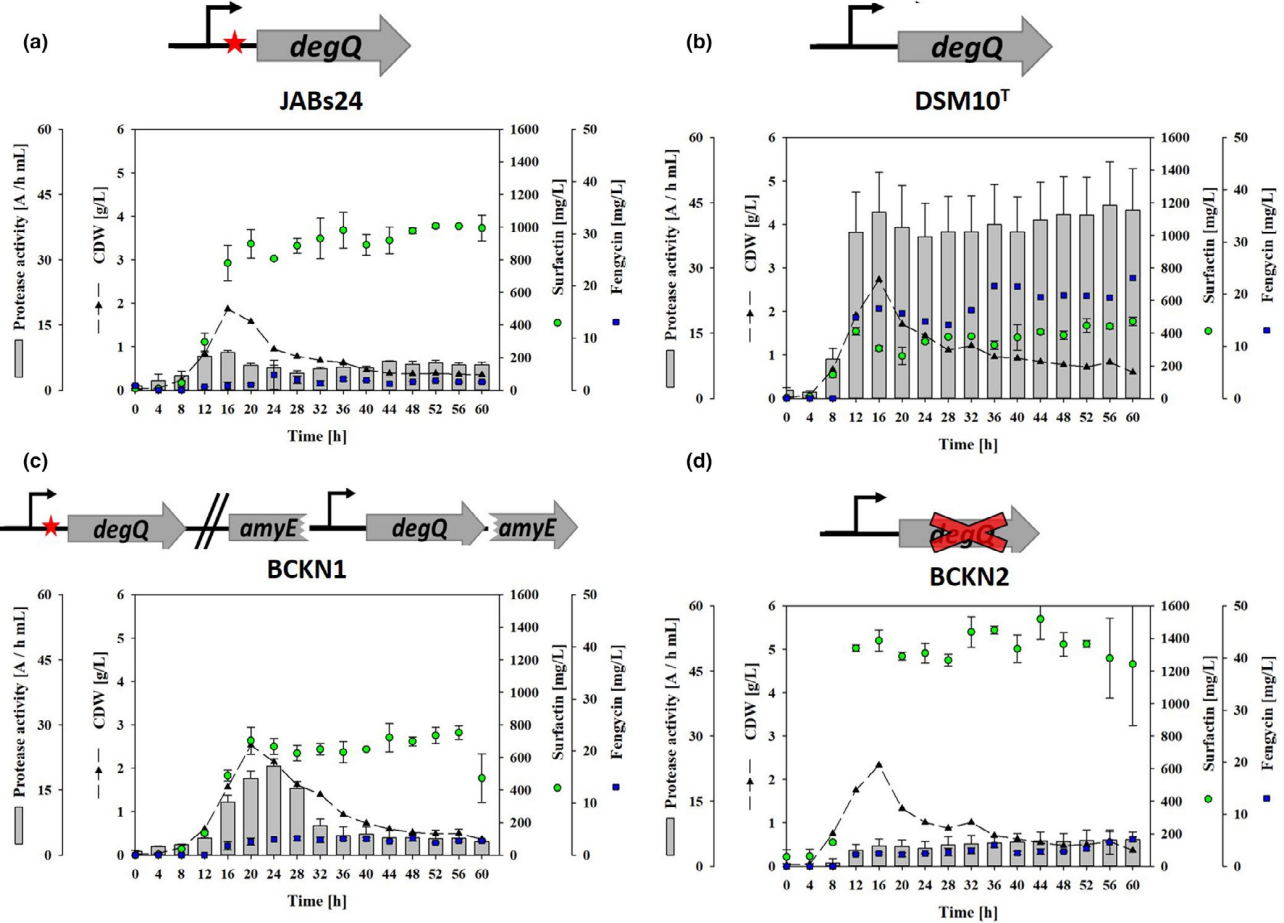
Comparison of lipopeptide production and extracellular protease activity during the time course of shake flask cultivation with 8 g/L glucose. Production parameters were determined for (a) JABs24 (168 *sfp*+), (b) DSM10^T^, (c) BCKN1 (JABs24 *amyE*::P*_degQ_*‐*degQ* from DSM10^T^), and (d) BCKN2 (DSM10^T^ *degQ*::*erm*). Gray bars indicate the extracellular protease activity, dashed lines represent the cell dry weight (CDW) and green dots display the surfactin, blue dots represent the plipastatin concentration over cultivation time. The data points represent a mean of at least two biological replicates. The error bars show the standard deviation of calculated values

Using 8 g/L glucose, cell dry weights reduced drastically after complete glucose depletion during the cultivation, resulting in detectable cell lysis without any stationary phase (Willenbacher et al., 2015). However, as previously shown by Vahidinasab et al., (2020), the concentrations of surfactin and plipastatin are not negatively affected by the reduction of cell dry weight (CDW) during glucose limitation. Specifically, the wild‐type strains JABs24 and DSM10^T^ showed contrary productivities with respect to surfactin and plipastatin formation (Figure 3a,b). While JABs24 produced a maximum surfactin concentration of 1007 mg/L, only 0.6 mg/L of plipastatin was detected (LOD and LOQ for plipastatin is 27 and 82 ng/zone, Geissler et al., 2017). In contrast, DSM10^T^ produced a comparatively low surfactin titer of 473 mg/L but 27 mg/L of plipastatin. In comparison, the corresponding *degQ* mutant strains showed altered lipopeptide productivities (Figure 3c,d). In the case of BCKN1, the strain JABs24 with native *degQ* expression, a reduced surfactin concentration of 753 mg/L was measured, whereas the plipastatin titer was increased to 4.1 mg/L. In contrast, BCKN2, the *degQ* deletion mutant of DSM10^T^, showed a promising increase in surfactin production up to 1520 mg/L, whereas a 5.2‐fold decrease in plipastatin production to 5.2 mg/L was determined.

Since *degQ* expression is also directly associated with secretory protease production, JABs24 and DSM10^T^ wild‐type strains as well as their *degQ* mutants were examined for their ability to form extracellular proteases. Therefore, endopeptidase activity was measured in cell‐free supernatants using an azocasein assay. In detail, JABs24 showed a basal activity for extracellular proteases during cultivation with a comparatively small increase to 8.7 ΔA/(h·mL) during the late exponential phase (Figure 3a). In contrast, strain DSM10^T^ showed the highest protease activity of up to 42.8 ΔA/(h·mL), which reached a plateau after 12 h of cultivation (Figure 3b). In respect of the stability of lipopeptides, no reduction of surfactin and plipastatin concentration was detected for both JABs24 and DSM10^T^ suggesting that secretion of proteases has an inferior impact on lipopeptide production.

In comparison, integration of a natively expressed *degQ* version from the DSM10^T^ strain into JABs24 increased secretory protease activity 2 times (17.6 ΔA/(h·mL) after 24 h) compared to JABs24 (Figure 3c). A comparably great effect was observed for BCKN2, resulting in a continuous basal level of up to 6.2 ΔA/(h·mL) at the end of cultivation (Figure 3d). In this way, deletion of the *degQ* gene in DSM10^T^ reduced extracellular protease activity sevenfold.

Table 3 gives an overall summary of the effect of *degQ* gene expression on the lipopeptide productivity and secretory protease formation of the wild‐type strains JABs24 and DSM10^T^ and their *degQ* mutant strains.

**TABLE 3:** Summary of parameters of cultivation with JABs24 and DSM10^T^ wild‐type strains and their inversed *degQ* mutant strains BCKN1 and BCKN2.

*B*. *subtilis* strains	End of exponential phase						
	Cultivation time [h]	CDW [g/L]	surfactin conc. [mg/L]	Y*_P_*_/_ *_X_*_, surfactin_ [mg/g]	plipastatin conc. [mg/L]	Y*_P_*_/_ *_X_*_, plipastatin_ [mg/g]	secretory protease activity [ΔA/h·mL]
JABs24	20	1.58	898.7	568.8	0.5	0.32	5.7
DSM10^T^	16	2.73	306.9	112.4	18.6	6.81	42.8
BCKN1	16	1.56	488.0	312.8	4.2	2.69	9.8
BCKN2	20	1.33	1290.0	969.9	3.2	2.41	4.7

## 4 DISCUSSION

Due to the point mutation within the *degQ* promoter region, *B*. *subtilis* JABs24, the lipopeptide‐forming derivative of *B*. *subtilis* 168, shows a drastically reduced *degQ* gene expression. This circumstance was already described by Stanley and Lazazzera (2005) and confirmed by using *lacZ* reporter strains for a time‐resolved comparison of the expression of the two *degQ* promoter versions until the transient growth phase (Figure 2). In this process, the wild‐type strain DSM10^T^ showed much higher P*_degQ_* promoter activity compared to JABs24. Since DegQ is directly involved in the activation of the DegU response regulator, it is reasonable to assume that DSM10^T^ also displays a more stimulated DegU regulation. The positive feedback regulation of DegU‐P on *degQ* gene expression amplifies the difference between JABs24 and DSM10^T^ in terms of P*_degQ_* promoter activity. The varying DegQ‐mediated activation of the DegU regulon was also observed by the detection of the lipopeptides surfactin and plipastatin as well as the formation of secretory proteases. Accordingly, a natively expressed *degQ* version reduced surfactin but increased plipastatin production, while a significantly higher extracellular protease activity was detected in the presence of the non‐mutated *degQ* promoter version.

While surfactin production is negatively affected by DegQ‐associated DegU regulation, increased plipastatin biosynthesis is achieved in the presence of native *degQ* expression (Miras & Dubnau, 2016; Vahidinasab et al., 2020). This opposing regulatory mechanism was transferable to both JABs24 and DSM10^T^. Accordingly, after integration of a natively expressed *degQ* version in JABs24, the resulted strain BCKN1 produced only approx. 75% of surfactin but eightfold increased plipastatin titers, while the elimination of *degQ* in DSM10^T^ (strain BCKN2) showed a threefold increase in surfactin concentration and a fivefold reduction in plipastatin formation. In summary, DegQ can be considered as a regulatory decision point for DegU‐mediated production of either surfactin or plipastatin. Accordingly, lipopeptide‐producing derivative strains of *B*. *subtilis* 168, encoding silenced *degQ* expression, appear to be predestinated for surfactin formation, whereas DSM10^T^ and other *B*. *subtilis* wild‐type strains show more effective plipastatin or fengycin production.

Besides the biotechnologically useful production of lipopeptides, another aspect is the DegU‐associated formation of secretory proteases. In this study, the comparison of extracellular protease activities between JABs24 and DSM10^T^ showed the effect of silenced *degQ* gene expression. Thus, DSM10^T^ was found to have fivefold higher protease activity compared to JABs24. Notably, both surfactin and plipastatin showed no decrease in their concentrations during the cultivation process, although secretory protease activity differed significantly between both strains, suggesting that lipopeptides are less targeted by native extracellular proteases. Subsequent integration of a natively expressed *degQ* version in JABs24 (strain BCKN1) increased extracellular protease production twofold, whereas a sevenfold decrease was observed after deletion of *degQ* in DSM10^T^ (BCKN2). Altogether, evidence for a quantitative effect of *degQ* expression on the production of extracellular proteases is documented.

## 5 CONCLUSIONS

The *degQ* loci of the lipopeptide‐producing strains DSM10^T^ and JABs24 differ by a single point mutation that leads to a drastic reduction of *degQ* gene expression in JABs24. Based on opposing regulatory mechanisms related to the DegU regulator, the presented strains show beneficial yields in surfactin or plipastatin production, which was confirmed by constructed *degQ* mutant strains. An additional negative effect of silenced *degQ* expression in JABs24 was furthermore quantitatively examined on the formation of extracellular proteases. Although a lipopeptide degradation cannot be excluded, different signal strengths of the protease activities measured during the cultivation processes did not lead to a decrease in lipopeptide concentration.

## 6 ETHICS STATEMENT

None required.

## CONFLICT OF INTERESTS

None declared.

## AUTHOR CONTRIBUTIONS

Lars Lilge: Conceptualization—Lead, Project administration—Lead, Investigation—Equal, Supervision—Lead, Data curation—Equal, Formal analysis—Equal, Methodology—Equal, Writing‐original draft—Equal, Writing‐review & editing—Equal. Maliheh Vahidinasab: Visualization—Leading, Data curation—Equal, Formal analysis—Equal, Methodology—Equal, Writing‐original draft—Equal, Writing‐review & editing—Equal. Isabel Adiek, Philipp Becker and Chanthiya Kuppusamy Nesamani: Methodology—Equal, Data curation—Equal, Formal analysis—Equal. Chantal Treinen: Formal analysis—Equal, Methodology—Equal, Writing‐review & editing—Equal. Mareen Hoffmann and Kambiz Morabbi Heravi: Formal analysis—Equal, Writing‐review & editing—Equal. Marius Henkel: Formal analysis—Equal, Data curation—Equal, Writing‐review & editing—Equal. Rudolf Hausmann: Funding acquisition—Leading, Formal analysis—Equal, Writing‐review & editing—Equal.

## Data Availability

All data generated or analyzed during this study are included in this published article. An overview of the collected data is available in Zenodo at https://doi.org/10.5281/zenodo.5511929
